# Supplementary material for: Genome-Wide Association Study of Fluorescent Oxidation Products Accounting for Tobacco Smoking Status in Adults from the French EGEA Study
Source: Antioxidants (Basel). 2022 Apr 20;11(5):802. doi: 10.3390/antiox11050802 (PMC9137810; doi:10.3390/antiox11050802)
Supplement: Supplementary file 1 [file antioxidants-11-00802-s001.zip › antioxidants-1622736-supplementary.pdf]

ONLINE SUPPLEMENTARY MATERIALS

Genome-wide association study of Fluorescent Oxidation Products accounting for tobacco smoking status in adults from the French EGEA study

Laurent Orsi, PhD [1]; Patricia Margaritte-Jeannin, PhD [2,‡]; Miora Andrianjafimasy, PhD [1,‡], Orianne Dumas, PhD [1]; Hamida Mohamdi, MSc [2]; Emmanuelle Bouzigon, MD, PhD [2]; Florence Demenais, MD [2]; Régis Matran, MD, PhD [3,4,5]; Farid Zerimech, MD, PhD [3,4,5]; Rachel Nadif, PhD [1,$]; Marie-Hélène Dizier, PhD [2,$]

Author Affiliations

[1] Université Paris-Saclay, UVSQ, Univ. Paris-Sud, INSERM, équipe d'Epidémiologie Respiratoire Intégrative, CESP, F-94807 Villejuif, France;

[2] Université Paris Cité, INSERM, UMR 1124, Group of Genomic Epidemiology and Multifactorial Diseases, F-75006 Paris, France

[3] Univ. Lille, ULR 4483 – IMPECS, F-59000 Lille, France

[4] CHU Lille, F-59000 Lille, France

[5] Institut Pasteur de Lille, F-59000 Lille, France

[‡] These authors contributed equally to the work

[$] Co-last authors

Supplement to "*2.1. Study population (EGEA study)*" section under MATERIALS AND METHODS. Description of the EGEA study

Figure S1. Flow chart of the studied population.

Figure S2. Manhattan plot of GWAS results of FlOPs levels (A) in the whole sample (n=1,216), (B) in never-smokers (n=604) and (C) in current smokers (n=275).

X-axis shows chromosome position and Y-axis shows –log10 P-values. The horizontal lines are drawn at $P = 1.3 \times 10^{-7}$ for genome-wide significant threshold.

Figure S3. Quantile-quantile (QQ) plot of GWAS results of FlOPs levels (A) in the whole sample (n=1,216), (B) in never-smokers (n=604) and (C) in current smokers (n=275).

Y-axis shows the -log10 of observed P-values. X-axis shows the -log10 of expected P-values.

Figure S4. Regional plot of association results using imputed genetic data for the three top-SNPs in the whole sample (n=1,216), region around rs270404 (A), region around rs13223298 (B) and region around rs491274 (C).

The left Y-axis shows the -log10 P-values. The position of the SNPs in megabases (GRCh 37.p13) is shown on the X-axis. Rs270404, rs13223298 and rs491274 are coloured in purple. The degree of LD (from the hg19/1000 Genomes reference panel) between all SNPs and the reference SNP (coloured in purple) we selected in the same region is reflected by the colour of the dots (red being the highest degree of LD).

Figure S5. Regional plot of association results using imputed genetic data for the three top-SNPs in never-smokers (n=604), region around rs17823624 (A), region around rs6606856 (B) and region around rs2962642 (C).

The left Y-axis shows the -log10 P-values. The position of the SNPs in megabases (GRCh 37.p13) is shown on the X-axis. Rs17823624, rs6606856 and rs2962642 are coloured in purple. The degree of LD (from the hg19/1000 Genomes reference panel) between all SNPs and the reference SNP (coloured in purple) we selected in the same region is reflected by the colour of the dots (red being the highest degree of LD).

Figure S6. Regional plot of association results using imputed genetic data for the three top-SNPs in current smokers (n=275), region around rs3851212 (A), region around rs1793958 (B) and region around rs17174795 (C).

The left Y-axis shows the -log10 P-values. The position of the SNPs in megabases (GRCh 37.p13) is shown on the X-axis. Rs3851212, rs1793958, rs6606856 and rs17174795 are coloured in purple. The degree of LD (from the hg19/1000 Genomes reference panel) between all SNPs and the reference SNP (coloured in purple) we selected in the same region is reflected by the colour of the dots (red being the highest degree of LD).

Table S1. Association between FlOPs level and characteristics of the studied population (n=1,216).

Table S2. Three top SNPs in the whole sample: Consistency of the results in two independent sub-samples.

Table S3. Three top SNPs in never-smokers and in current smokers: consistency of the results in two independent sub-samples.

Table S4. Results from eQTL browser Phenoscanner v2.

Table S5. meQTLs for the three top-SNPs identified in GWAS in the whole sample, in never-smokers and in current smokers.

Table S6. Regulatory elements for the three top-SNPs (and proxies with r^2>0.80) identified in GWAS in the whole sample, in never-smokers and in current smokers.

Supplement to "2.1. *Study population (EGEA study)*" section under MATERIALS AND METHODS

Description of the EGEA study

The EGEA study is a cohort based on an initial group of asthma cases recruited in chest clinics from Grenoble, Lyon, Marseille, Montpellier and Paris, along with their first-degree relatives, and a group of controls (1991-1995, EGEA1, https://egeanet.vjf.inserm.fr/index.php/en/). The protocol and descriptive characteristics have been described previously [1,2]. Briefly, the asthma cases and their first-degree relatives were recruited from respiratory or allergic clinics. The adult cases were recruited in the five cities and children cases were recruited in Paris, Grenoble and Marseille. Control adults were recruited from electoral rolls in Paris, Lyon, Montpellier and Grenoble, a check-up centre in Marseille and surgery clinic from the same hospital in Paris and Grenoble. Control children were always recruited from surgery clinics. An overall matching by month of exam, age decade, sex and centre was done. A 12-year follow-up of the initial cohort was conducted between 2003 and 2007 (EGEA2). Among the alive cohort (n=2002), 92% (n=1845) completed a short self-administered questionnaire, and among them 1602 (n=1571 adults aged ≥16 years) had a complete examination including lung function tests, skin prick test to 12 aeroallergens, and blood samples. Inclusion criteria were based on standardized procedures (including four questions from validated and standardized British Medical Research Council/European Coal and Steel Community, American Thoracic Society, and European Community Respiratory Health Survey (ECRHS) questionnaires: '*Have you ever had attacks of breathlessness at rest with wheezing?*', '*Have you ever had asthma attacks?*'; If yes: '*Was this diagnosis confirmed by a physician?*' and '*Have you had an asthma attack in the last 12*

months?') to diagnose asthma and to determine respiratory and allergic symptoms, treatments, environmental exposures, and lifestyle characteristics.

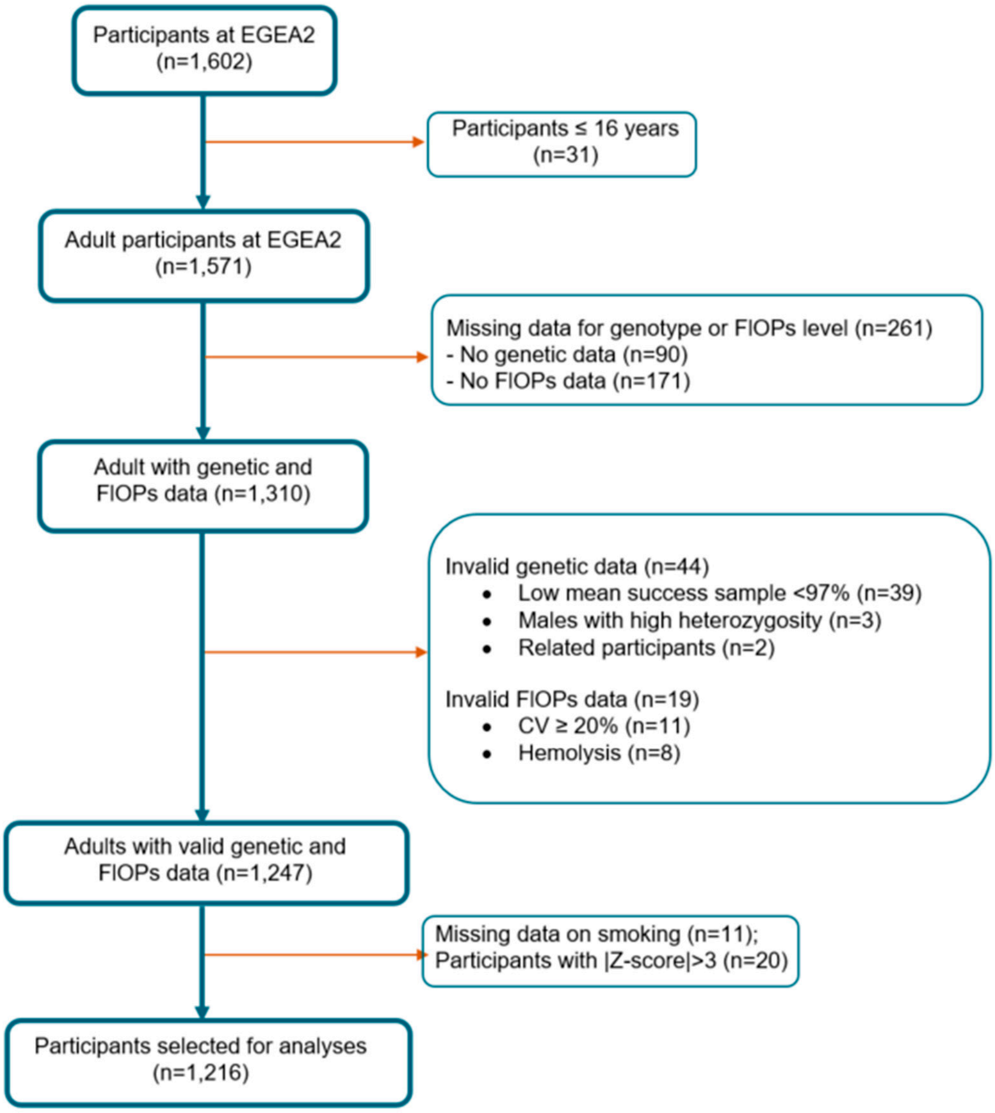

Figure S1. Flow chart of the studied population

A

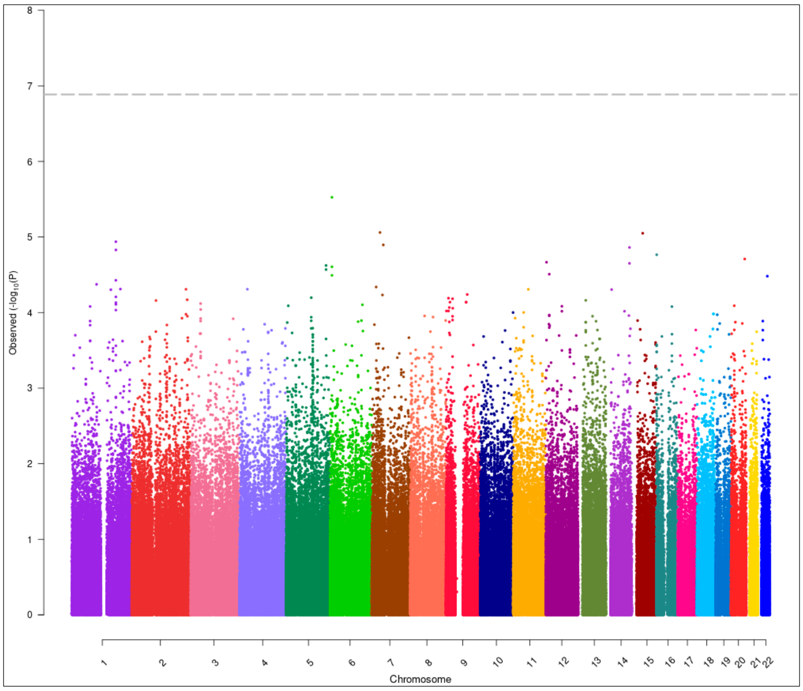
Observed (-log10(P))
8
7
6
5
4
3
2
1
0
1 2 3 4 5 6 7 8 9 10 11 12 13 14 15 16 17 18 19 20 21 22
Chromosome

B

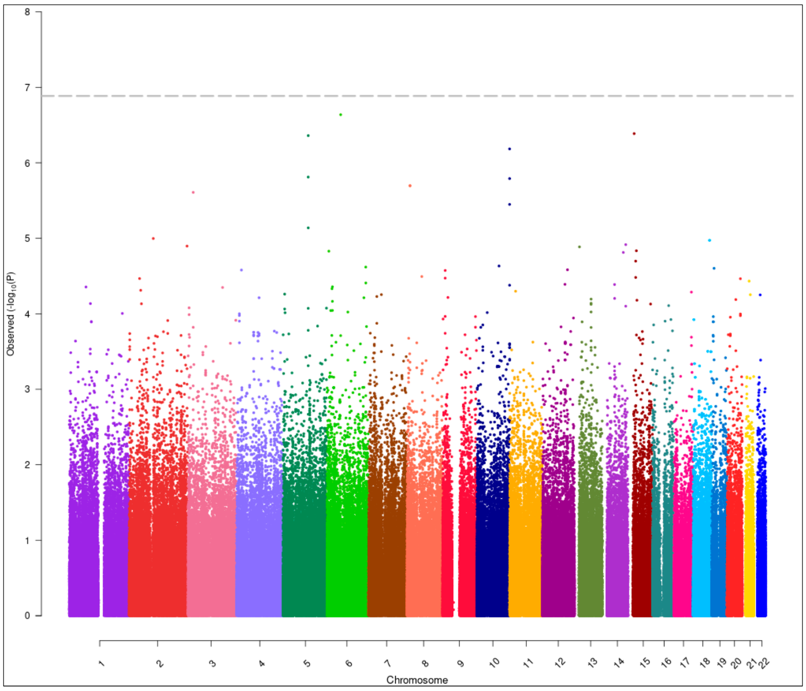
Observed (-log10(P))
8
7
6
5
4
3
2
1
0
1 2 3 4 5 6 7 8 9 10 11 12 13 14 15 16 17 18 19 20 21 22
Chromosome

C

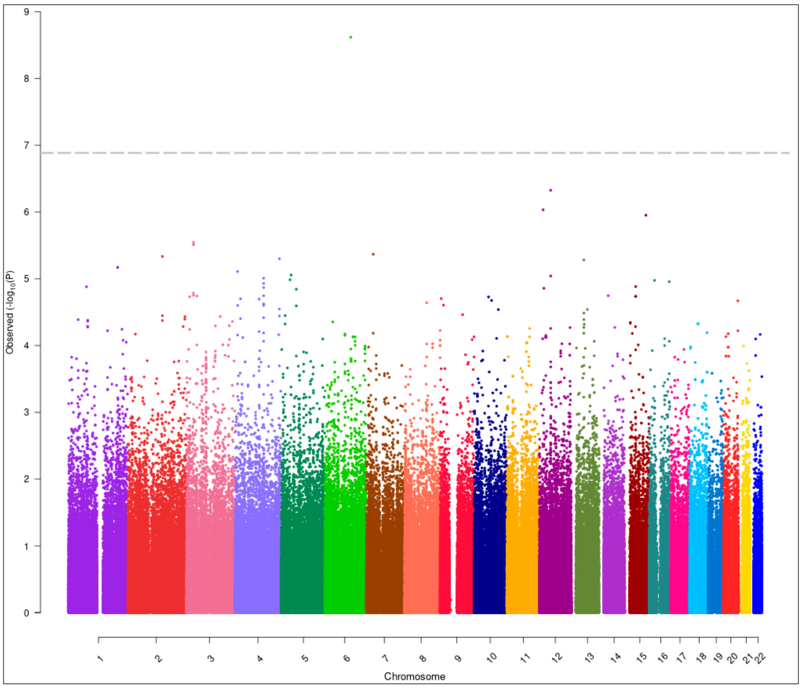

Figure S2. Manhattan plot of GWAS results of FlOPs levels (A) in the whole sample (n=1,216), (B) in never-smokers (n=604) and (C) in current smokers (n=275).

X-axis shows chromosome position and Y-axis shows –log10(P-value). The horizontal lines are drawn at P = 1.3 X 10^{-7} for genome-wide significant threshold.

A

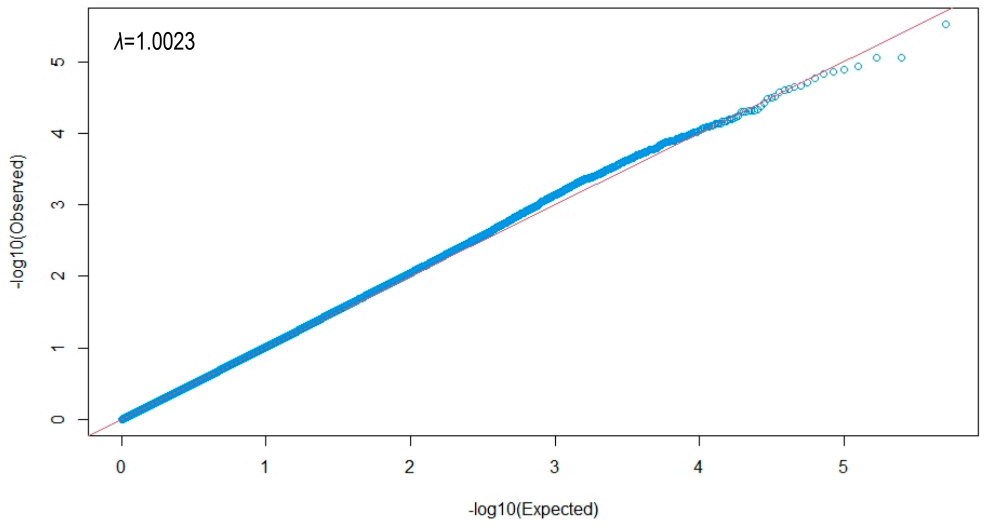

B

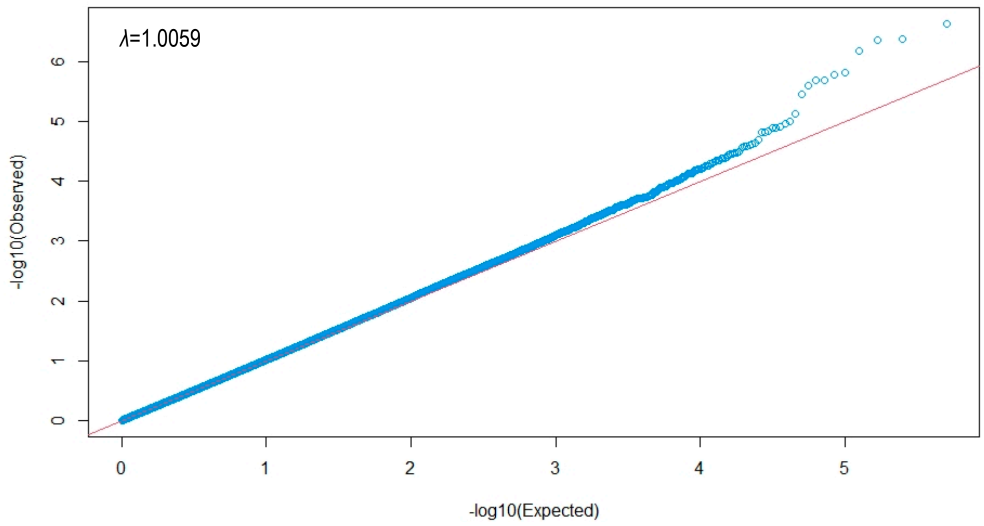

C

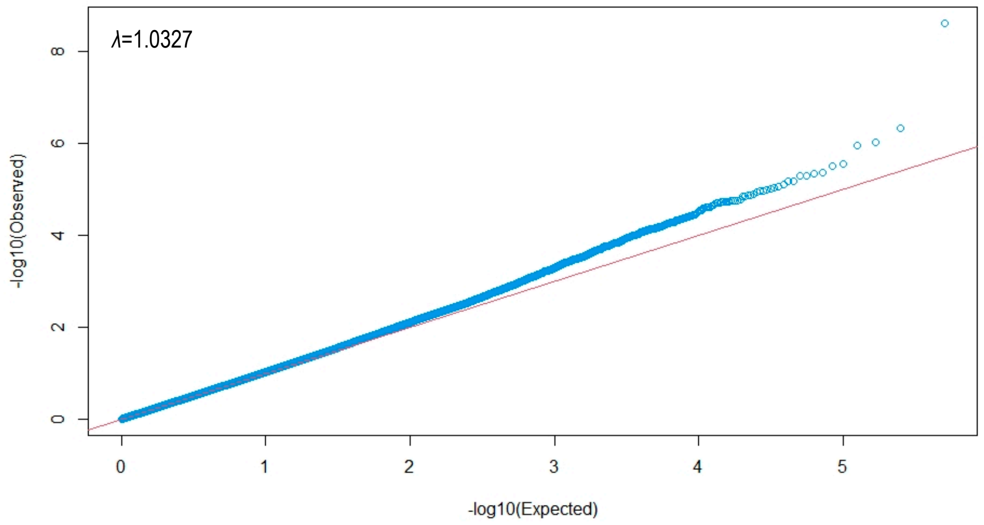

Figure S3. Quantile-quantile (QQ) plot of GWAS results of FlOPs levels (A) in the whole sample (n=1,216), (B) in never-smokers (n=604) and (C) in current smokers (n=275).

Y-axis shows the -log10 of observed P-values. X-axis shows the -log10 of expected P-values.

A

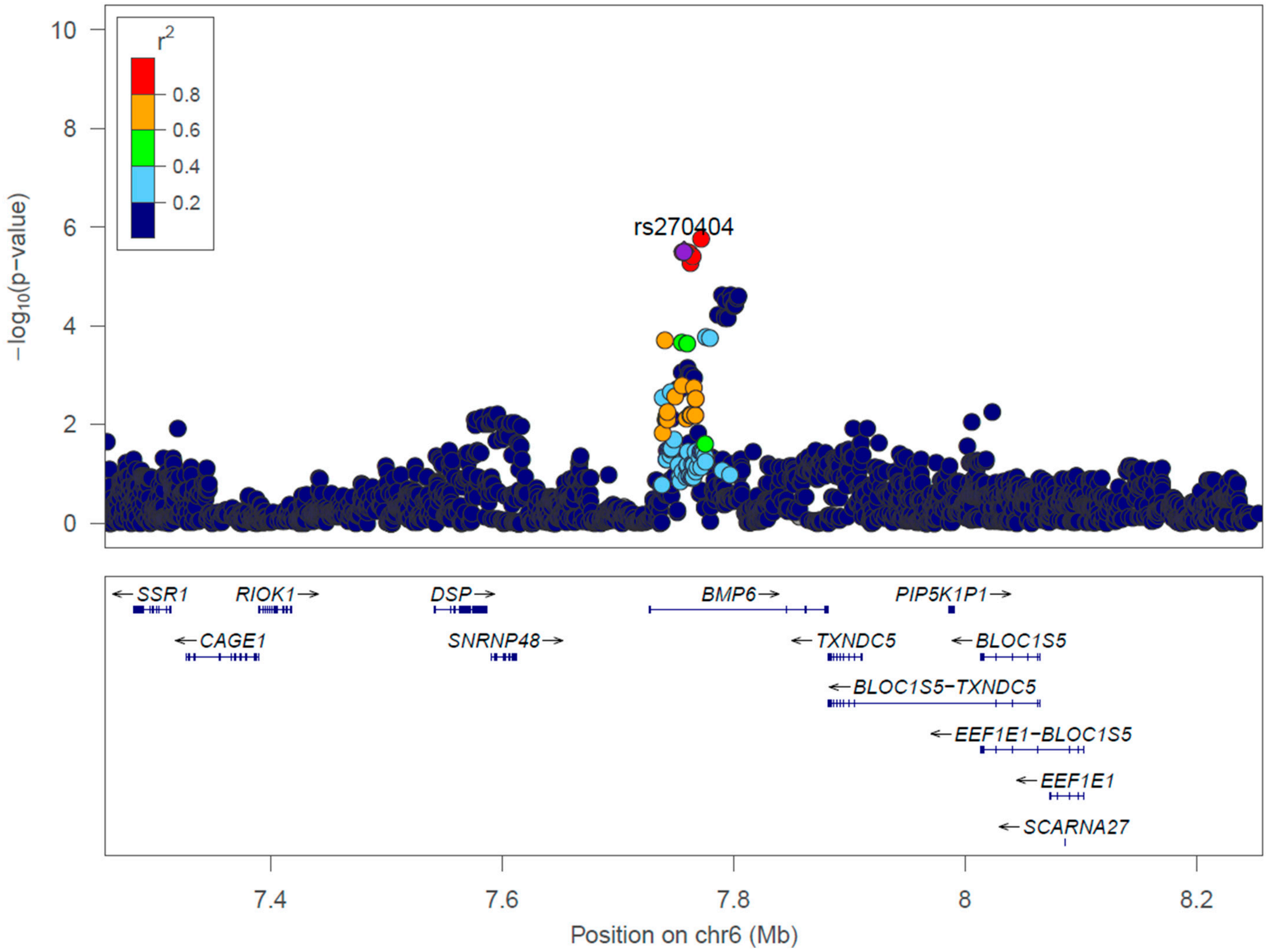

r2
0.8
0.6
0.4
0.2
10
8
6
4
2
0
−log10(p−value)
rs270404
SSR1
RIOK1
DSP
BMP6
PIP5K1P1
CAGE1
SNRNP48
TXNDC5
BLOC1S5
BLOC1S5−TXNDC5
EEF1E1−BLOC1S5
EEF1E1
SCARNA27
7.4
7.6
7.8
8
8.2
Position on chr6 (Mb)

B

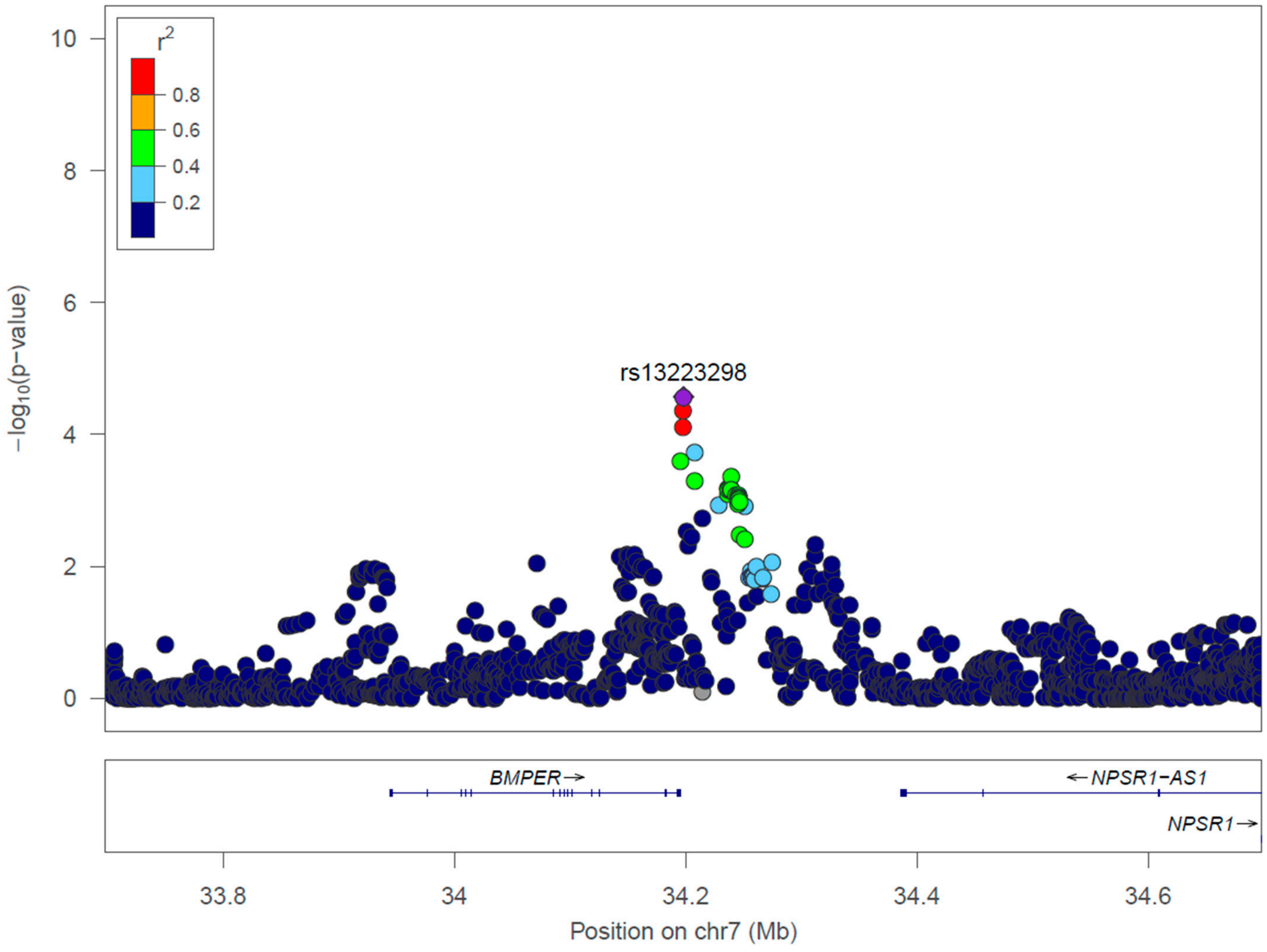

r2
0.8
0.6
0.4
0.2
10
8
6
4
2
0
−log10(p−value)
rs13223298
BMPER→
←NPSR1−AS1
NPSR1→
33.8
34
34.2
34.4
34.6
Position on chr7 (Mb)

C

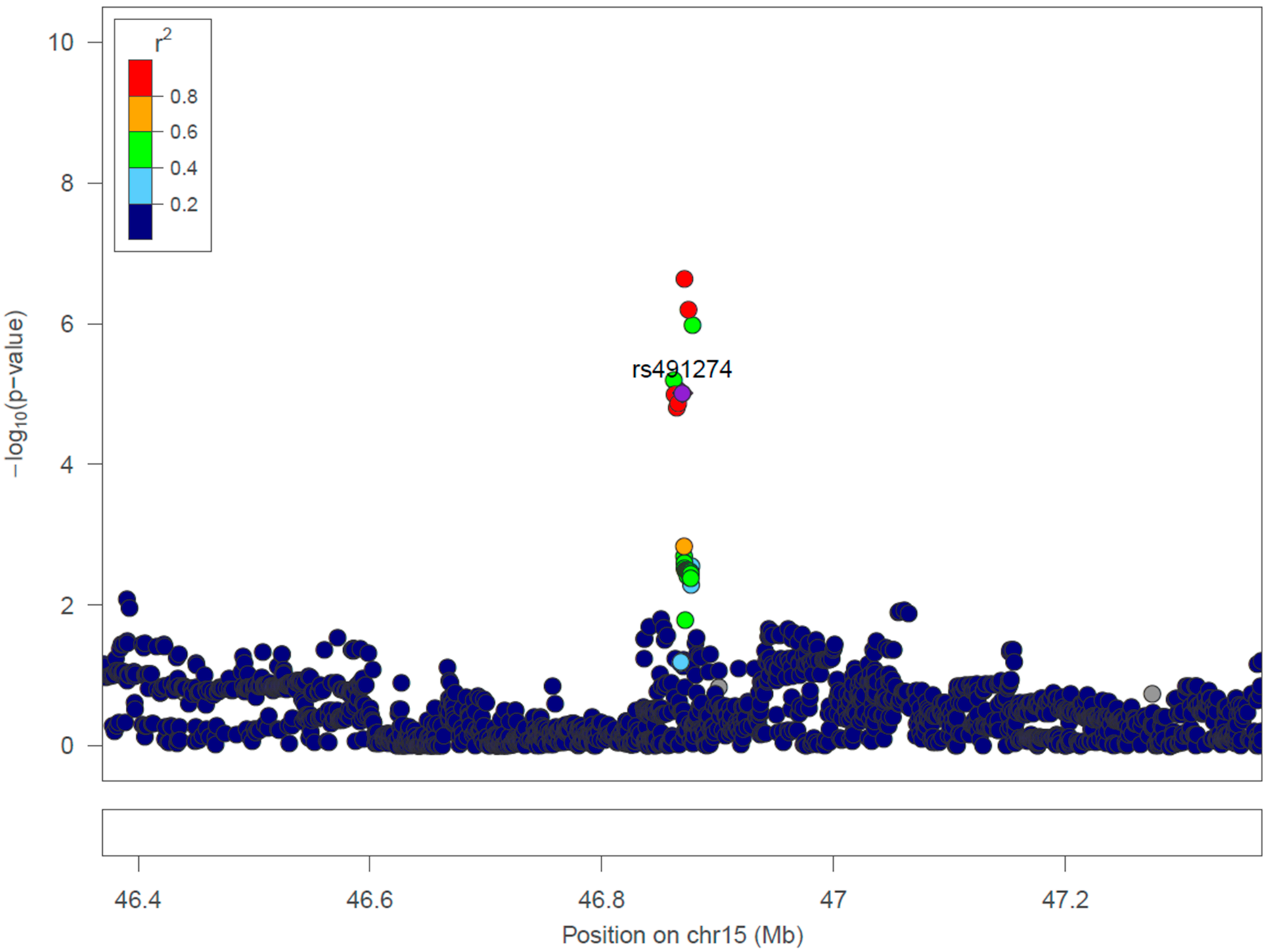

Figure S4. Regional plot of association results using imputed genetic data for the three top-SNPs in the whole sample (n=1,216), region around rs270404 (A), region around rs13223298 (B) and region around rs491274 (C).

The left Y-axis shows the -log10 P-values. The position of the SNPs in megabases (GRCh 37.p13) is shown on the X-axis.
Rs270404, rs13223298 and rs491274 are coloured in purple. The degree of LD (from the hg19/1000 Genomes reference panel) between all SNPs and the reference SNP (coloured in purple) we selected in the same region is reflected by the colour of the dots (red being the highest degree of LD).

A

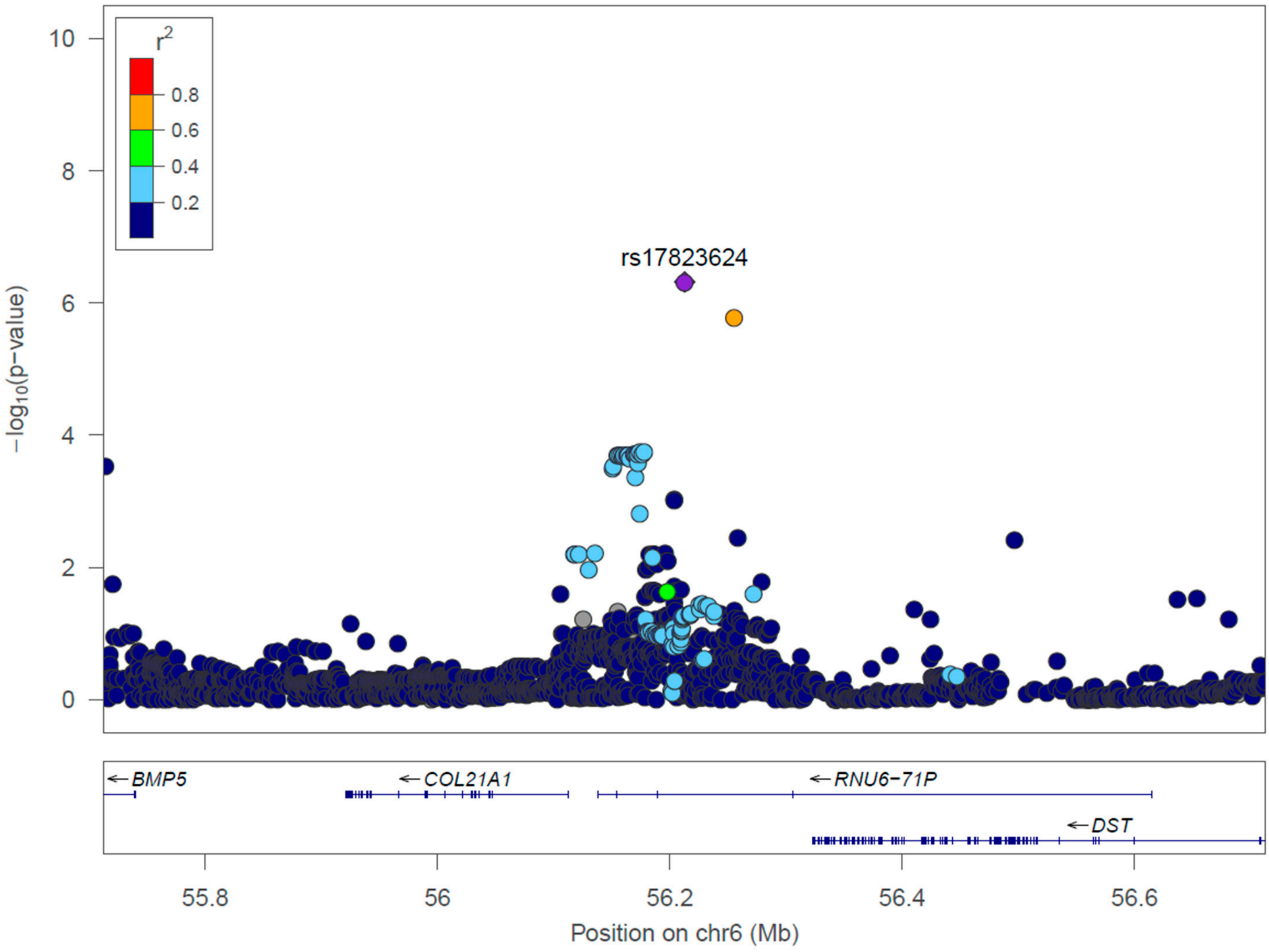
r2
0.8
0.6
0.4
0.2
rs17823624
10
8
6
4
2
0
−log10(p−value)
BMP5
COL21A1
RNU6−71P
DST
55.8
56
56.2
56.4
56.6
Position on chr6 (Mb)

B

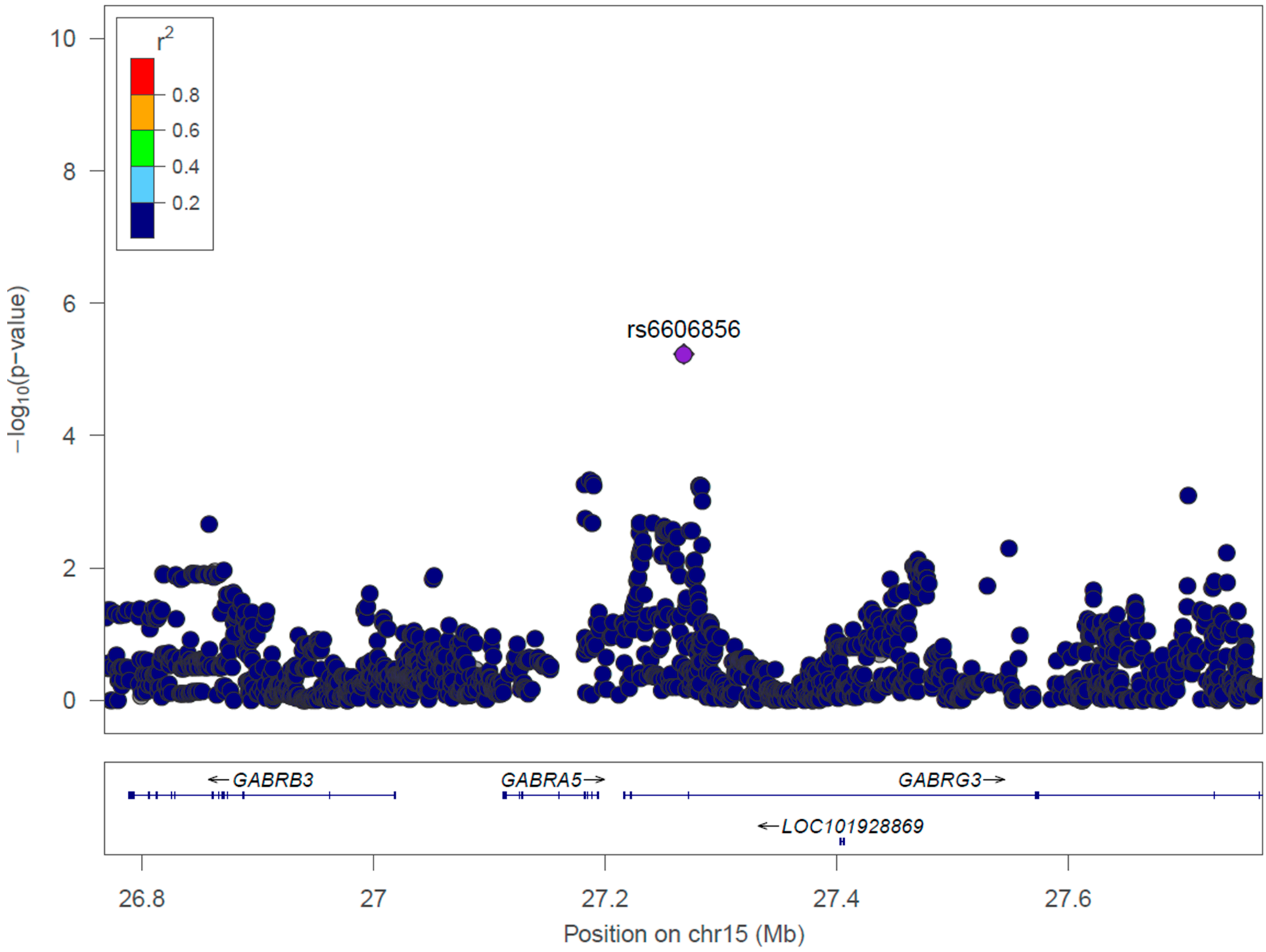

r2
0.8
0.6
0.4
0.2
10
8
6
4
2
0
−log10(p−value)
rs6606856
←GABRB3
GABRA5→
GABRG3→
←LOC101928869
26.8
27
27.2
27.4
27.6
Position on chr15 (Mb)

C

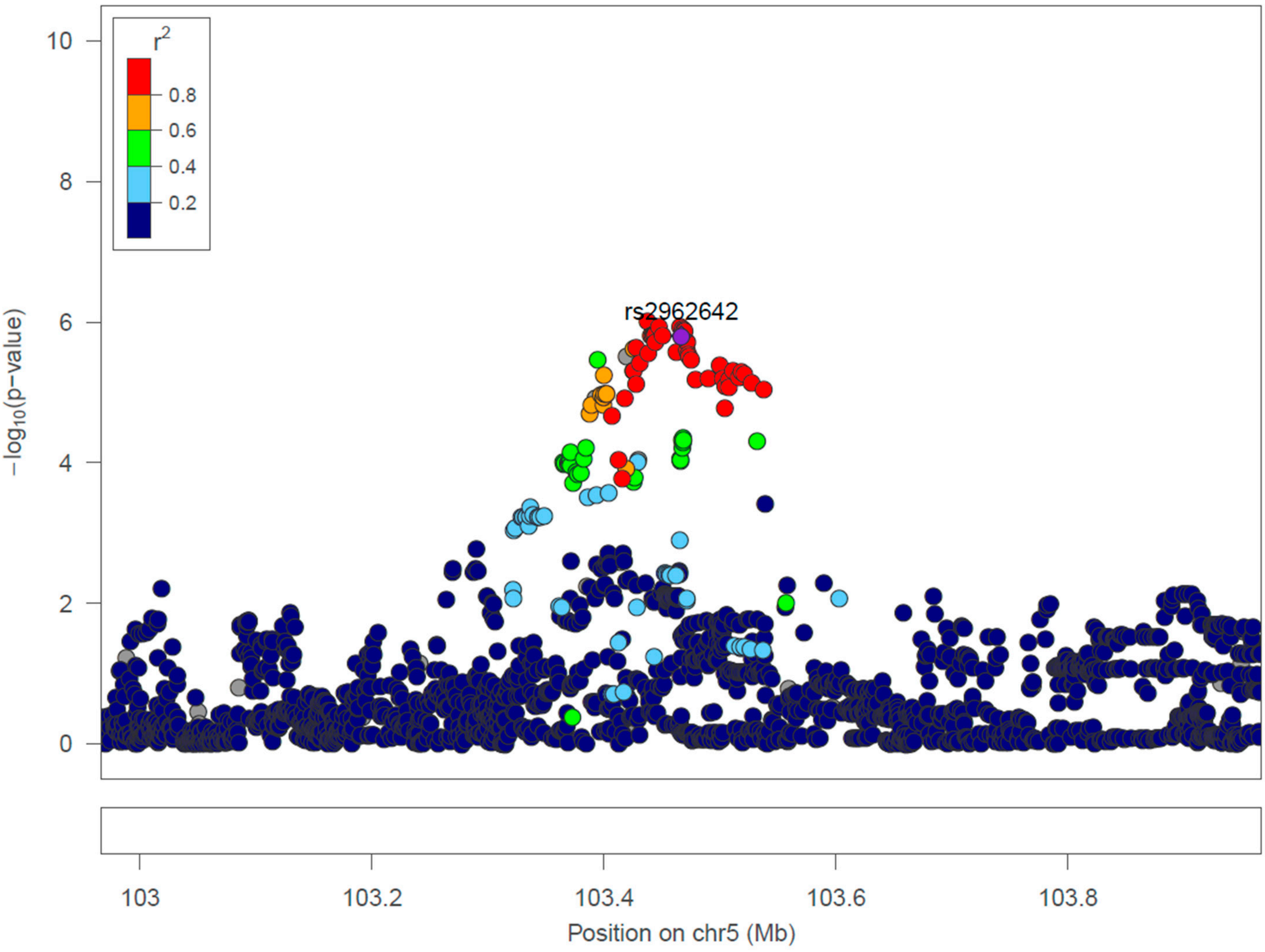

Figure S5. Regional plot of association results using imputed genetic data for the three top-SNPs in never-smokers (n=604), region around rs17823624 (A), region around rs6606856 (B) and region around rs2962642 (C).

The left Y-axis shows the -log10 P-values. The position of the SNPs in megabases (GRCh 37.p13) is shown on the X-axis.

Rs17823624, rs6606856 and rs2962642 are coloured in purple. The degree of LD (from the hg19/1000 Genomes reference panel) between all SNPs and the reference SNP (coloured in purple) we selected in the same region is reflected by the colour of the dots (red being the highest degree of LD).

A

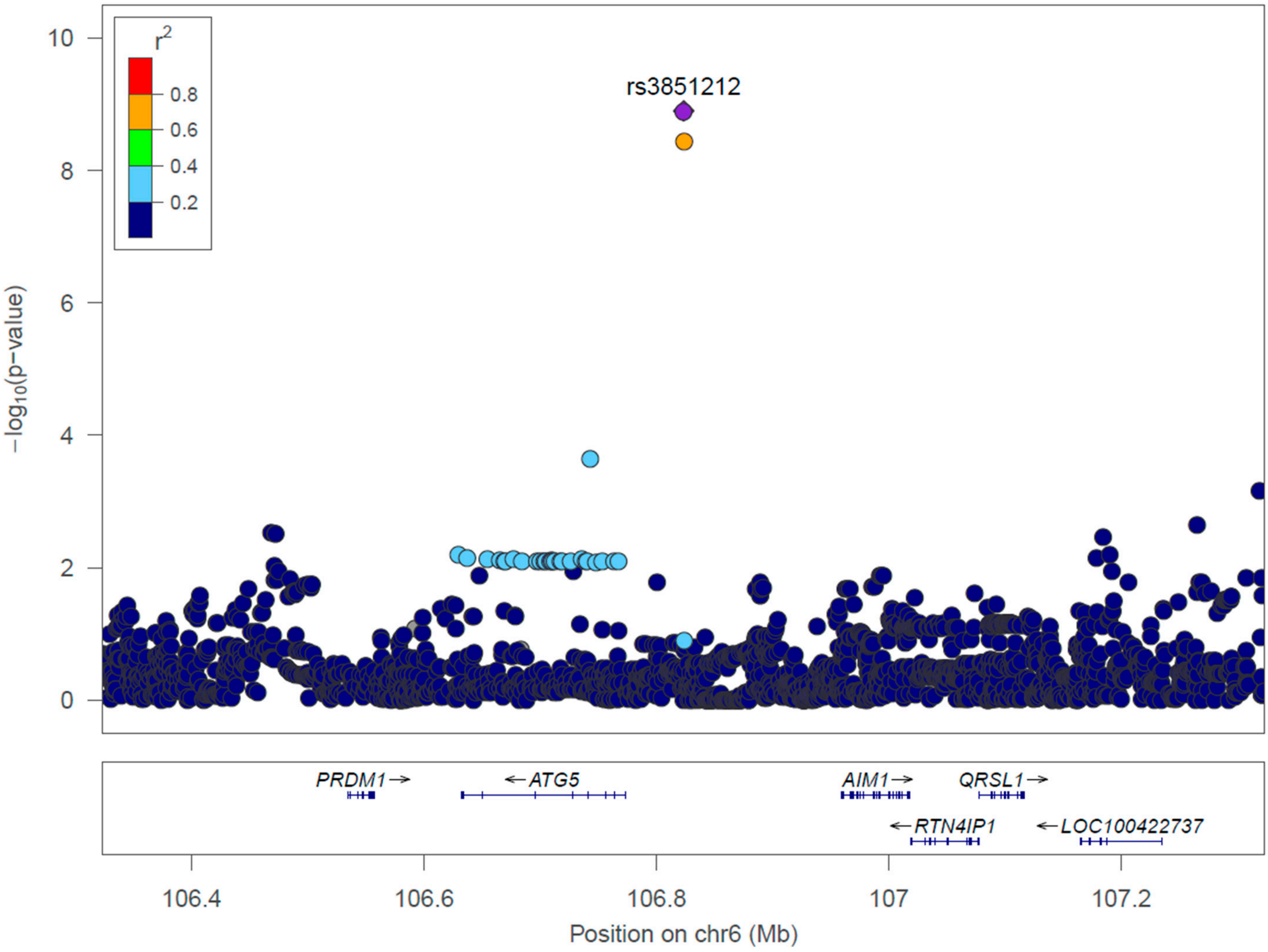

rs3851212
r2
0.8
0.6
0.4
0.2
10
8
6
4
2
0
−log10(p−value)
PRDM1→
←ATG5
AIM1→
QRSL1→
←RTN4IP1
←LOC100422737
106.4
106.6
106.8
107
107.2
Position on chr6 (Mb)

B

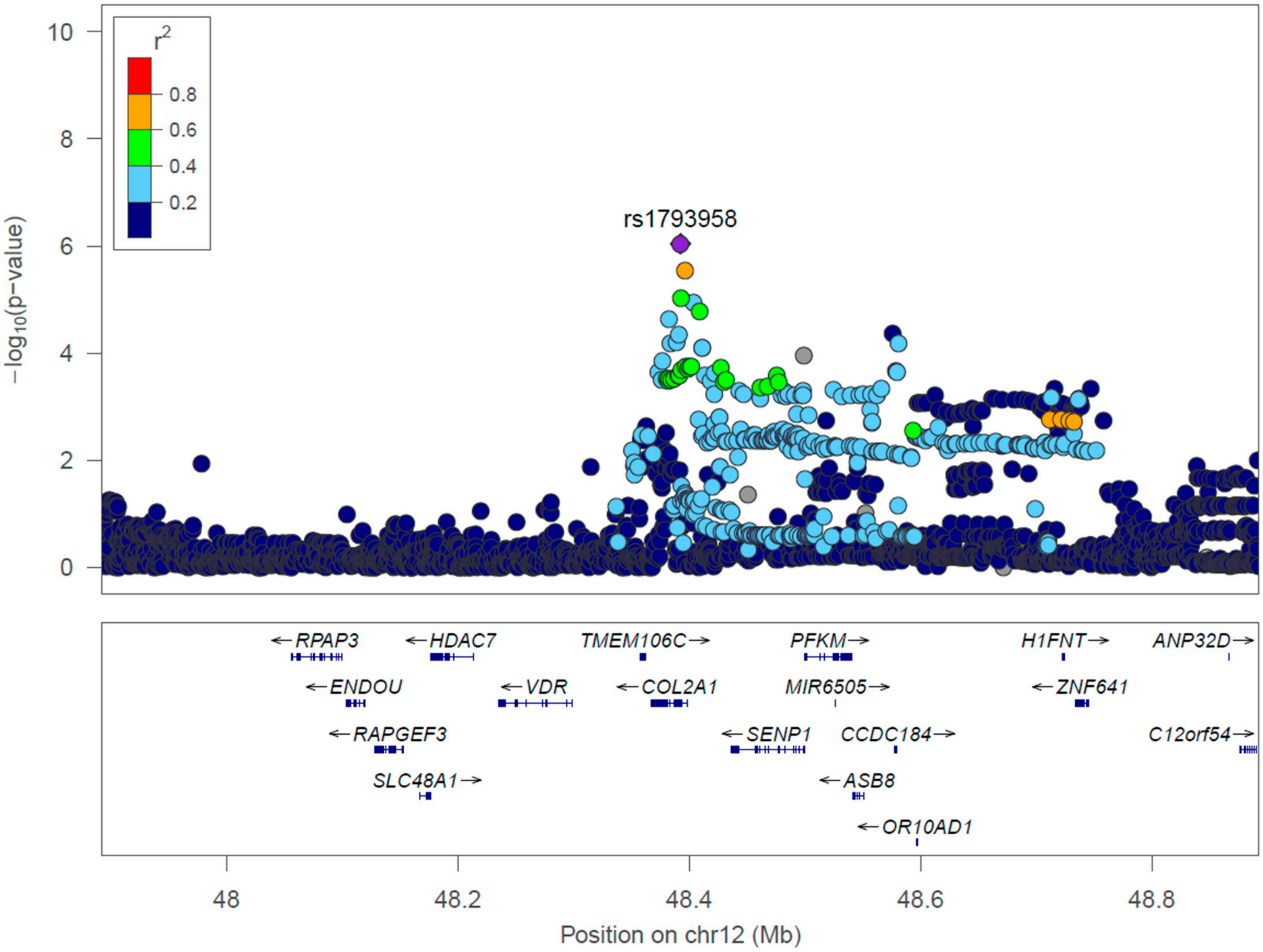

r2
0.8
0.6
0.4
0.2
rs1793958
10
8
6
4
2
0
−log10(p−value)
RPAP3
HDAC7
TMEM106C
PFKM
H1FNT
ANP32D
ENDOU
VDR
COL2A1
MIR6505
ZNF641
RAPGEF3
SENP1
CCDC184
C12orf54
SLC48A1
ASB8
OR10AD1
48
48.2
48.4
48.6
48.8
Position on chr12 (Mb)

C

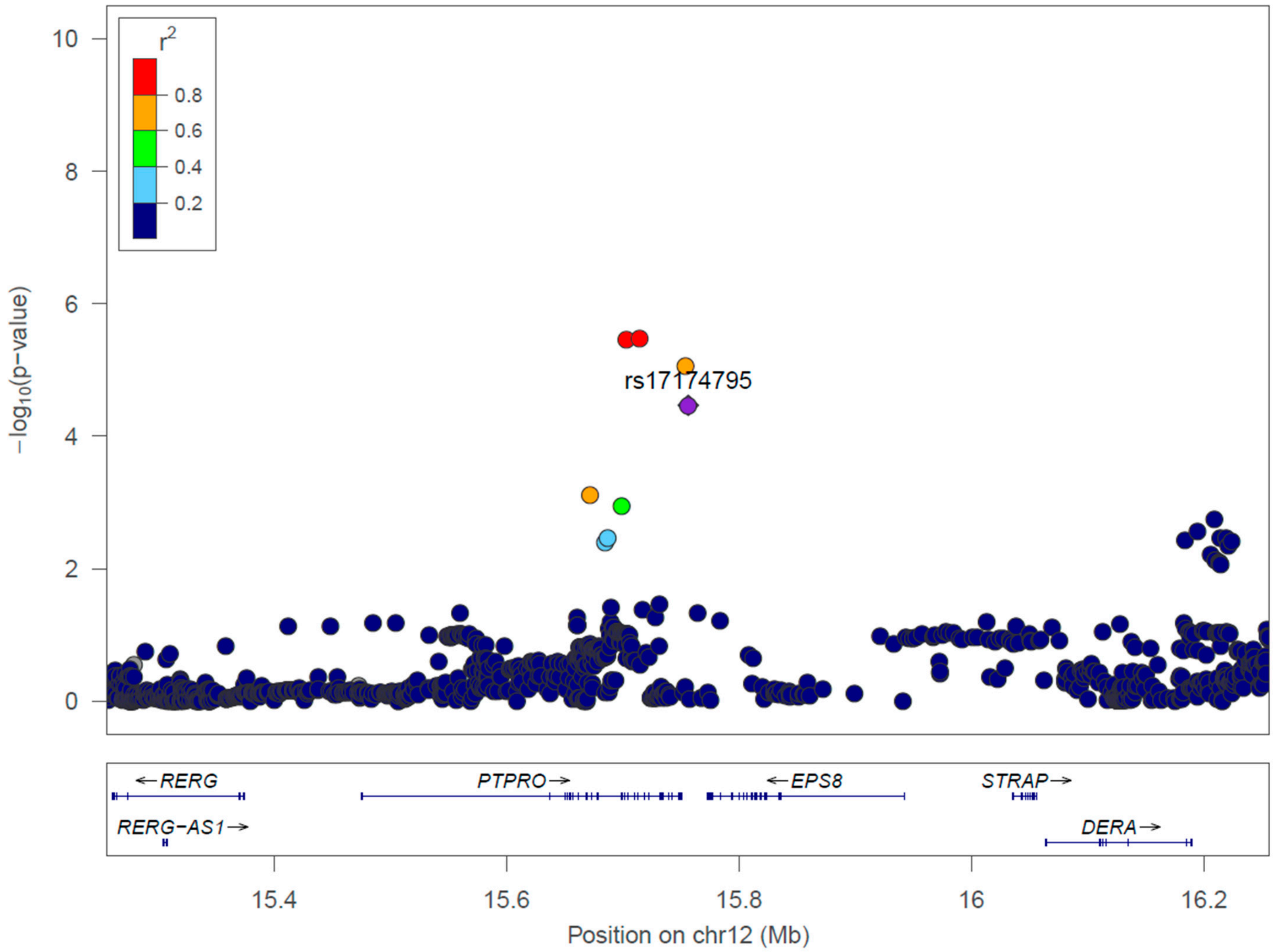

Figure S6. Regional plot of association results using imputed genetic data for the three top-SNPs in current smokers (n=275), region around rs3851212 (A), region around rs1793958 (B) and region around rs17174795 (C).

The left Y-axis shows the -log10 P-values. The position of the SNPs in megabases (GRCh 37.p13) is shown on the X-axis.

Rs3851212, rs1793958, rs6606856 and rs17174795 are coloured in purple. The degree of LD (from the hg19/1000 Genomes reference panel) between all SNPs and the reference SNP (coloured in purple) we selected in the same region is reflected by the colour of the dots (red being the highest degree of LD).

Table S1. Association between FlOPs level and characteristics of the studied population (n=1,216)

	Whole sample (n=1,216)	Levels of FlOPs GM (Q1, Q3)	*Adjusted-p*
FlOPs, RFU/mL, GM (Q1, Q3)	92.3 (80, 105)		
Age, m ± sd	43.3 ± 16.4	ϱ=0.30	*<1.0 X 10^{-4}*
Sex, n (%)			*5.0 X 10^{-3}*
Men	595 (48.9)	91.4 (79, 103)	
Women	621 (51.1)	93.2 (81, 107)	
Current smoking status[a], n (%)			*<1.0 X 10^{-4}*
Never-smokers	604 (49.7)	89.1 (77, 101)	
Ex-smokers	337 (27.7)	97.2 (85, 111)	
Current smokers	275 (22.6)	93.8 (82, 107)	
Smoking, pack-year, n (%)			*<1.0 X 10^{-4}*
Never smokers	604 (49.7)	89.1 (77, 101)	
<10	384 (31.6)	93.4 (81, 107)	
10-20	111 (9.1)	98.7 (88, 108)	
> 20	117 (9.6)	100.3 (88, 114)	
Neutrophils, cell/mm^3, m ± sd	3,986 ± 1,391	ϱ= 0.07	*0.10*
FEV_1≥80%, n (%)			*0.29*
No	100 (8.3)	98.1 (84, 112)	
Yes	1,097 (91.7)	91.8 (80, 105)	

Adjusted *p*-value of association were estimated using generalized linear regression of log-FlOPs, adjusted for age (continuous), sex and smoking pack-year (pack-year), taking into account within family correlation; GM, geometric mean; Q1, 1st quartile; Q3, 3rd quartile; m, mean; sd, standard deviation; ϱ, pearson correlation; FEV_1, Forced Expiratory Volume in one second;

[a], all pairwise comparisons *p*-values (scheffe's test) were < 0.05

Table S2. Three top SNPs in the whole sample: Consistency of the results in two independent sub-samples

Chr	Gene	Nearest Gene	Genomic location	Marker	Position bp (hg38)	A1/A2	EAF	Controls (n=243)		Cases/Relatives (n=973)		Homogeneity test	
								beta ± se	*p*	beta ± se	*p*	khi²	*p*
6	*BMP6*	*TXNDC5*	Intronic	rs270404	7 757 141	A/G	0.41	-0.16 ± 0.09	*0.08*	-0.21 ± 0.05	*1.1 X 10⁻⁵*	0.24	*0.62*
7		*BMPER*	3'-UTR	rs13223298	34 158 658	G/T	0.08	-0.32 ± 0.19	*0.09*	-0.34 ± 0.08	*5.6 X 10⁻⁵*	0.01	*0.92*
15		*SEMA6D*	Intergenic	rs491274	46 577 328	A/G	0.09	-0.30 ± 0.15	*0.05*	-0.30 ± 0.08	*8.8 X 10⁻⁵*	0.00	*1.00*

Chr, Chromosome; A1/A2, baseline/effect allele; EAF, Effect allele frequency estimated from the reference panel 1000G (European population).
Beta and standard error (se) were estimated using Gaussian linear model taking into account EGEA family structure and adjusted for principal components

Table S3. Three top SNPs in never-smokers and in current smokers: consistency of the results in two independent sub-samples

								Controls		Cases/Relatives		Homogeneity test	
Chr	Gene	Nearest Gene	Genomic location	Marker	Position bp (hg38)	A1/A2	EAF	beta ± se	*p*	beta ± se	*P*	khi²	*p*
	Never-smokers							n=114		n=490			
6	*COL21A1*	*DST*	Intronic	rs17823624	56 348 021	A/G	0.92	0.46 ± 0.18	1.1×10^{-2}	0.44 ± 0.09	4.9×10^{-6}	0.10	*0.91*
15	*GABRG3*	*GABRA5*	Intronic	rs6606856	27 022 887	C/T	0.70	-0.29 ± 0.13	2.6×10^{-2}	-0.28 ± 0.06	9.2×10^{-6}	0.06	*0.81*
5		*NUDT12*	Intergenic	rs2962642	104 131 010	A/G	0.24	0.24 ± 0.13	6.6×10^{-2}	0.31 ± 0.07	2.8×10^{-6}	0.20	*0.65*
	Current smokers							n=59		n=216			
6	*CRYBG1*	*ATG5*	Intronic	rs3851212	106 375 664	A/G	0.94	-0.75 ± 0.30	1.5×10^{-2}	-0.85 ± 0.15	3.7×10^{-8}	0.07	*0.79*
12	*COL2A1*	*TMEM106C*	Intronic	rs1793958	47 998 650	A/G	0.61	-0.43 ± 0.18	1.9×10^{-2}	-0.41 ± 0.09	8.9×10^{-6}	0.00	*0.97*
12		*PTPRO*	Intergenic	rs17174795	15 603 555	G/T	0.87	-0.93 ± 0.40	2.5×10^{-2}	-0.58 ± 0.13	2.1×10^{-5}	0.72	*0.40*

Chr, Chromosome; A1/A2, baseline/effect allele; EAF, Effect allele frequency estimated from the reference panel 1000G (European population).
Beta and standard error (se) were estimated using Gaussian linear model taking into account EGEA family structure and adjusted for principal components

Table S4. Results from eQTL browser Phenoscanner v2 (http://www.phenoscanner.medschl.cam.ac.uk/)

Chr	Marker	Position bp (hg38)	Ancestry	Tissue	Gene expression	*p*	Study	Reference
GWAS in never-smokers								
6	rs17823624	56 348 021	European	Whole blood	*DST*	2.0×10^{-15}	eQTLGen	[3]
GWAS in current smokers								
12	rs1793958	47 998 650	European	Whole blood	*OR10AD1*	1.3×10^{-21}	eQTLGen	[3]
12	rs1793958	47 998 650	European	Whole blood	*PFKM*	3.7×10^{-41}	eQTLGen	[3]
12	rs1793958	47 998 650	European	Whole blood	*SENP1*	2.3×10^{-8}	eQTLGen	[3]
12	rs1793958	47 998 650	European	Whole blood	*TMEM106C*	1.7×10^{-11}	eQTLGen	[3]
12	rs1793958	47 998 650	European	Whole blood	*VDR*	1.1×10^{-19}	eQTLGen	[3]

Chr, Chromosome.

References

1. Kauffmann, F.; Dizier, M.H.; Annesi-Maesano, I.; Bousquet, J.; Charpin, D.; Demenais, F.; Ecochard, D.; Feingold, J.; Gormand, F.; Grimfeld, A.; et al. EGEA (Epidemiological Study on the Genetics and Environment of Asthma, Bronchial Hyperresponsiveness and Atopy)-- Descriptive Characteristics. *Clin Exp Allergy* **1999**, *29 Suppl 4*, 17–21.
2. Kauffmann, F.; Dizier, M.H. EGEA (Epidemiological Study on the Genetics and Environment of Asthma, Bronchial Hyperresponsiveness and Atopy)--Design Issues. EGEA Co-Operative Group. *Clin Exp Allergy* **1995**, *25 Suppl 2*, 19–22, doi:10.1111/j.1365-2222.1995.tb00413.x.
3. Võsa, U.; Claringbould, A.; Westra, H.-J.; Bonder, M.J.; Deelen, P.; Zeng, B.; Kirsten, H.; Saha, A.; Kreuzhuber, R.; Kasela, S.; et al. Unraveling the Polygenic Architecture of Complex Traits Using Blood EQTL Metaanalysis. *bioRxiv* **2018**, 447367, doi:10.1101/447367.
